# A Rapid and Convenient Method for Fluorescence Analysis of *In Vitro* Cultivated Metacestode Vesicles from *Echinococcus multilocularis*

**DOI:** 10.1371/journal.pone.0118215

**Published:** 2015-02-23

**Authors:** Zhe Cheng, Fan Liu, Shan Zhu, Huimin Tian, Liang Wang, Yanhai Wang

**Affiliations:** 1 State Key Laboratory of Cellular Stress Biology, School of Life Sciences, Xiamen University, Xiamen, Fujian, China; 2 Medical College, Xiamen University, Xiamen, Fujian, China; Beijing Forestry University, CHINA

## Abstract

We here describe a convenient method for preparation, fixation and fluorescence analysis of *in vitro* cultivated metacestode vesicles from *E. multilocularis*. Parasite materials could be prepared in one hour, did not need to be sectioned, and were subsequently utilized for further whole-mount staining assays directly. Using these preparations, in combination with conventional fluorescence staining techniques, we could detect the expression and subcellular localization of a specific protein and identify *in situ* proliferative or apoptotic cells in the germinal layer of metacestode vesicles. Based on this approach, future molecular and cellular analysis of *Echinococcus* metacestode vesicles in the *in vitro* system will be greatly facilitated.

## Introduction

The metacestode larval stage of the fox-tapeworm *Echinococcus multilocularis* is the causative agent of alveolar echinococcosis (AE), which is considered to be one of the most lethal helminthic infections in humans [1]. An infection is initiated when the intermediate host (rodents, humans) ingests infective eggs which contain the oncosphere larval. The parasite then develops in the liver into cyst-like metacestode vesicles, which grow cancer-like and infiltrate the tissue of the host, forming new vesicles and even metastases. The metacestode vesicles are bounded by a thin cellular layer (germinal layer, GL) and filled by hydatid cyst fluid. The laminated layer (LL), an acellular, carbohydrate-rich sheath secreted by the underlying GL, protects the GL from host cells and/or host extracellular matrix [2]. The GL buds towards the inside giving rise to brood capsules, which in turn generate protoscoleces. After ingestion of the protoscoleces by the definitive host (canids), they attach to the intestine and develop into the adult tapeworms which produce eggs.

*E. multilocularis* has recently emerged as a laboratory model for elucidating underlying molecular mechanisms of host-parasite interactions and larval taeniid cestode development, largely due to its accessibility to *in vitro* cultivation [3]. The cultivation system comprises the co-cultivation of *E. multilocularis* metacestode vesicles and host feeder cells, an axenic cultivation system for metacestode vesicles, and a system by which metacestode vesicles are generated *in vitro* from primary cell cultures [4]. Therefore, molecular and cellular analysis of the vesicles cultured *in vitro* is fundamental and necessary.

The morphological and structural characteristics of *E. multilocularis* metacestode vesicles lead to some limitations when a conventional immunostaining (or other methods using macromolecular labeling / detecting materials) is performed in the GL of the vesicles. The LL protects the GL from host cells and also protects the GL from contacting the antibody. Penetration of the antibody during immunohistological staining usually can be improved by sectioning, which is, however, inconvenient for the *in vitro* cultivated vesicles. The bladder-like vesicles are filled with cyst fluid inside, normally single in the *in vitro* system and almost inevitably broken, collapsed and folded during the embedding and dehydrating processes. As a result, the GL and LL of the vesicles are easily twisted and overlapped in the slices, leading to inconvenience in the subsequent observation. This problem could be solved in a certain extent by pre-embedding vesicles in agarose (experience in our laboratory), which complicates the experiment process and needs substantial experience in subsequent sectioning. Allowing long incubation times with the antibody is another way to improve antibody penetration through the whole-mount immunostaining technique (samples do not need to be sectioned), whereas it usually prolongs the staining procedure to several days and may increase the non-specific background. Recently, Klaus Brehm’s research group has successfully developed an effective method for whole-mount immunofluorescence analysis of *in vitro* cultivated *E. multilocularis* vesicles [5, 6]. The vesicles were opened manually using a syringe tip to allow the antibody and other staining reagents to enter into the vesicles and contact the GL cells, avoiding long incubation times for the penetration of antibody through the LL. Nevertheless, this method needs delicate and time-consuming operation and may not be appropriate for manipulation of a large amount of vesicles and small vesicles. Simple and rapid sample preparation methods that facilitate the staining of protein / nucleic acid in the GL of vesicles are therefore still needed.

Here we developed a convenient method of vesicle sample preparation for fluorescence analysis. We anticipate that this method will improve analysis of cellular process of *in vitro* cultivated *Echinococcus* metacestode vesicles, including protein expression and localization, cell proliferation and cell death.

## Materials and Methods

### Ethics statement

Animal experiments were performed in strict accordance with China regulations on the protection of experimental animals (Regulations for the Administration of Affairs Concerning Experimental Animals, version from July-18-2013) and specifically approved by the Institutional Animal Care and Use Committee of Xiamen University (Permit Number: 2013–0053). The mice were raised and housed at the Xiamen University Laboratory Animals Center (XMULAC) under standardised light-controlled conditions (lights on from 7:00 am to 7:00 pm) at room temperature (24±1°C) and 50±10% humidity, with free access to food and water. The protocol was in strict accord with good animal practice as defined by XMULAC. All animal experiments adhere to the ARRIVE Guidelines for reporting animal research [7]. A completed ARRIVE guidelines checklist is included in S1 ARRIVE Checklist.

### Vesicle culture and whole-mount sample preparation

*E. multilocularis* metacestode vesicles used in this study were *in vitro* cultured as described before [8]. Briefly, *in vivo* propagation of the parasite material was performed using mongolian jirds (*Meriones unguiculatus*) and ICR mice. Mice were infected intraperitoneally with 0.5 mL of parasite suspension and sacrificed using CO2 after 4–6 months of infection. Metacestodes tissue was immediately isolated from the infected mice and co-cultured with host feeder cells for about 8 weeks. Then the axenic cultivation of metacestode vesicles was performed under anaerobic and reducing conditions using conditioned medium that had previously been incubated in the presence of host cells.

The method of whole-mount sample preparation for fluorescence analysis is described in detail and discussed in the Results and Discussion section.

### Immunofluorescence staining and assays of cell proliferation and cell death

Immunofluorescence staining and detection of proliferative or apoptotic cells were all performed in whole-mount vesicles prepared as described above.

Immunofluorescence staining was performed by following a standard protocol as described by the antibody manufacturer. Briefly, vesicles were washed three times for 5 minutes in PBS and blocked in blocking buffer (PBS with 5% normal goat serum and 0.5% Triton X-100) for 30 minutes at room temperature. Samples were incubated overnight at 4°C with polyclonal rabbit anti-beta tubulin (CST, Cat. No. 2146S) or polyclonal rabbit anti-ERK1/2 [pTpY185/187] (Life Technologies, Cat. No. 44680G) antibodies diluted 1:250 in antibody dilution buffer (PBS with 1% BSA and 0.5% Triton X-100) and detected with Alexa 488 goat anti-rabbit antibodies (CST, Cat. No. 4412S) diluted 1:1000. DNA was counterstained with 1 μg/ml 4’, 6-diamidino-2-phenylindole (DAPI, Sigma) for 10 minutes at room temperature.

For detection of proliferative cells, a method based on the incorporation of 5-ethynyl-2′-deoxyuridine (EdU) was explored [9]. Vesicles were pre-incubated with EdU (Life Technologies) for 2 hours in the culture media and then prepared as described in the Results and Discussion section. The samples were then processed for detection of EdU-positive cells with Click-iT EdU Imaging Kit (Life Technologies, Cat. No. C10338), following the protocol as described by the manufacturer. DNA was counterstained with Hoechst 33342 for 10 minutes at room temperature.

For detection of apoptotic cells, a method based on the TdT-mediated dUTP nick end labeling (TUNEL) was explored. Vesicles were pretreated with 5 mM H2O2 for 4 hours to introduce apoptosis and then prepared as described in the Results and Discussion section. Apoptosis analysis was performed using *in situ* cell death detection kit (Roche, Cat. No. 11684809910). Deoxyribonuclease (DNase) treatment (Fermentas, 300 U/mL, 10 minutes at room temperature) was performed for the positive control according to the kit instruction manual. In permeabilisation step, samples were treated with either proteinase K (2 μg / mL) for 10 minutes or 0.3% Triton X-100 in 0.1% sodium citrate for 10 minutes at 20°C. After the TUNEL reaction, samples were counterstained with DAPI and then directly analyzed by fluorescence microscopy.

All of the fluorescence staining was imaged with a Nikon 80i fluorescence microscope. Contrast and light adjusting and merge of pictures were done by using Nikon image software supplied by the microscope manufacturer or Adobe Photoshop 8.0 software.

## Results and Discussion

Analysis of the cellular processes of *in vitro* cultivated *E. multilocularis* metacestode vesicle using fluorescence techniques has been technically difficult due to the characteristics of the structure of the vesicle. To improve this, we developed a rapid and convenient method of sample preparation for whole-mount fluorescence analysis.

The *in vitro* cultivated vesicles were washed in phosphate buffer saline (PBS), manually picked up and mounted onto a poly-L-lysine coated glass slide with pipettes. The vesicles and liquid were spread out with the pipette to keep individual vesicle from overlapping. Since subsequent steps in our protocol do not need complicated operations, we usually manipulate 20–30 vesicles on one slide at the same time. Most of the PBS was then wicked off and 50 μL of freshly prepared 4% paraformaldehyde (PFA) in PBS were immediately added for fixation. Another poly-L-lysine coated glass slide (or a 24 x 50 mm poly-L-lysine coated coverslip) was mounted onto the vesicles and gently tapped to break them. Vesicles were fixed at room temperature for 15–30 minutes and most of the PFA was wicked off after fixation. This step can not be omitted since redundant fluid causes slides sliding and a failure in following “freeze crack” step. The preparations were frozen at -80°C for 10 minutes and kept at room temperature for 15–20 seconds and then the two poly-L-lysine coated slides were immediately separated with a single-edged razor blade. During this step, one should keep the slides in the air. Do not put them on a bench, otherwise they will thaw very fast and the “freeze crack” will not work well. The “freeze crack” is the key step of the whole protocol, which is designed to quickly make cracks on the surface of the vesicle, allowing the antibody or other labeling / detecting reagents to enter into the vesicle and reach the germinal layer. The highlight of this step is that in most cases a single vesicle can be separated into two parts and each part firmly sticks to one poly-L-lysine coated slide. Therefore the GL and LL of the vesicles do not overlap and the samples are rarely lost from the slides during following permeabilization and washing steps. Consequently, the GL cells are entirely exposed and can be analyzed as typically done for the human cancer cells cultured in multi-well plates or on coverslips.

Taken together, our method has some advantages for the whole-mount sample preparation. First, the entire experimental process is fast, dose not need complicated operation and can be done in less than one hour. It also avoids long incubation times with the antibody which may increase the non-specific background. Second, the vesicles do not need to be opened manually and therefore the manipulation of a large amount of vesicles and the analysis of very small vesicles (diameter < 1 mm) would be much facilitated. Third, overlap of the GL and LL of the vesicles often happens in the slices using the traditional sectioning methods, whereas it rarely occurs using our method and thus the following observation is much more convenient. The last, the GL cells can be analyzed as cancer cells cultured on the coverslips with no further optimized steps.

**Fig 1:**
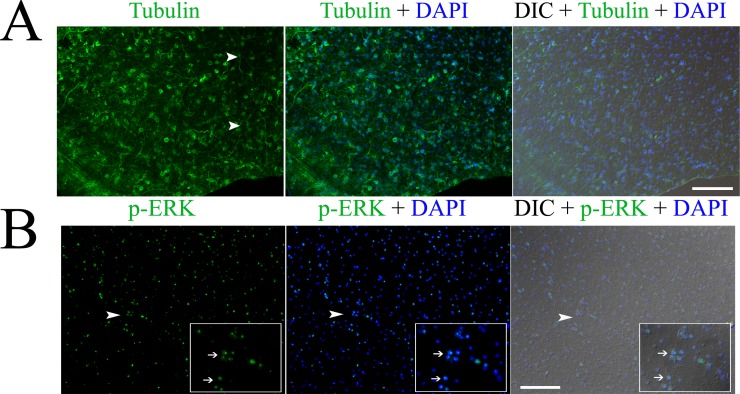
Immunofluorescence detection of beta tubulin (A) and phosphorylated ERK (p-ERK) (B) in the germinal layer of the whole-mount preparations of *in vitro* cultured *E. multilocularis* vesicles. Arrow heads in (A) indicate the neuron cells with long neurites. Arrow heads in (B) indicate the areas which are magnified in the inserts. Note the higher expression of p-ERK in the nucleolus of some cells indicated by the arrows in the inserts. Merges of the differential interference contrast (DIC) bright field microscopy with the fluorescence channels are shown. Bar = 125 μm.

Using these whole-mount preparations, we successfully performed immunofluorescence (IF) analysis in the GL cells. In the fixation step for IF, PFA concentration and fixation time is important and should be different for different antibody staining. In principle, higher PFA concentration and longer fix time resulted in better structure but worse staining, while lower concentration and shorter fix time led to brighter staining but worse structure. For most of the antibodies we have tested in our laboratory, 2–4% of PFA and 10 min-1 h of fix time usually brought good results. After the “freeze crack” step, a treatment of the whole-mount prepared samples with pre-chilled 95% ethanol for 1 min or 100% methanol for 5 min is optional. 95% ethanol sometimes can improve the fluorescence signal. However, it may also cause high background. Methanol is considered to minimize the background. We suggest that one should try this optional step when detecting a protein for the first time and then determine if this treatment is necessary. Using our protocol, we could detect the expression of beta tubulin, the major constituent of microtubules (Fig. 1A). Some neuron cells with long neurites could also be identified as described before (arrow heads in Fig. 1A) [5]. We also detected the phosphorylated form of EmMPK1, an ERK-like MAP kinase (Fig. 1B), which was previously shown to be dominantly localized in the nucleus of metacestodes of *E. multilocularis* (results based on the immunehistochemistry method using the parasite material directly collected from the host) [10].

Surprisingly, we also observed higher expression of phosphorylated ERK in the nucleolus of some GL cells (inserts in Fig. 1B). ERK has been implicated in the induction of rRNA synthesis [11], for which the localization in the nucleolus might indeed be expected and detected [12]. The decreased phosphorylation of ERK in the vesicles cultured in the serum free medium were also observed (data not shown), consisting with the fact that activation of *E. multilocularis* ERK is mediated by the host growth factors [10]. In addition, we successfully detected other proteins which we are interested in by using either commercial antibodies or self-prepared antiserum. Taken together, these results validate our method for IF analysis of *in vitro* cultivated *E. multilocularis* vesicles.

**Fig 2:**
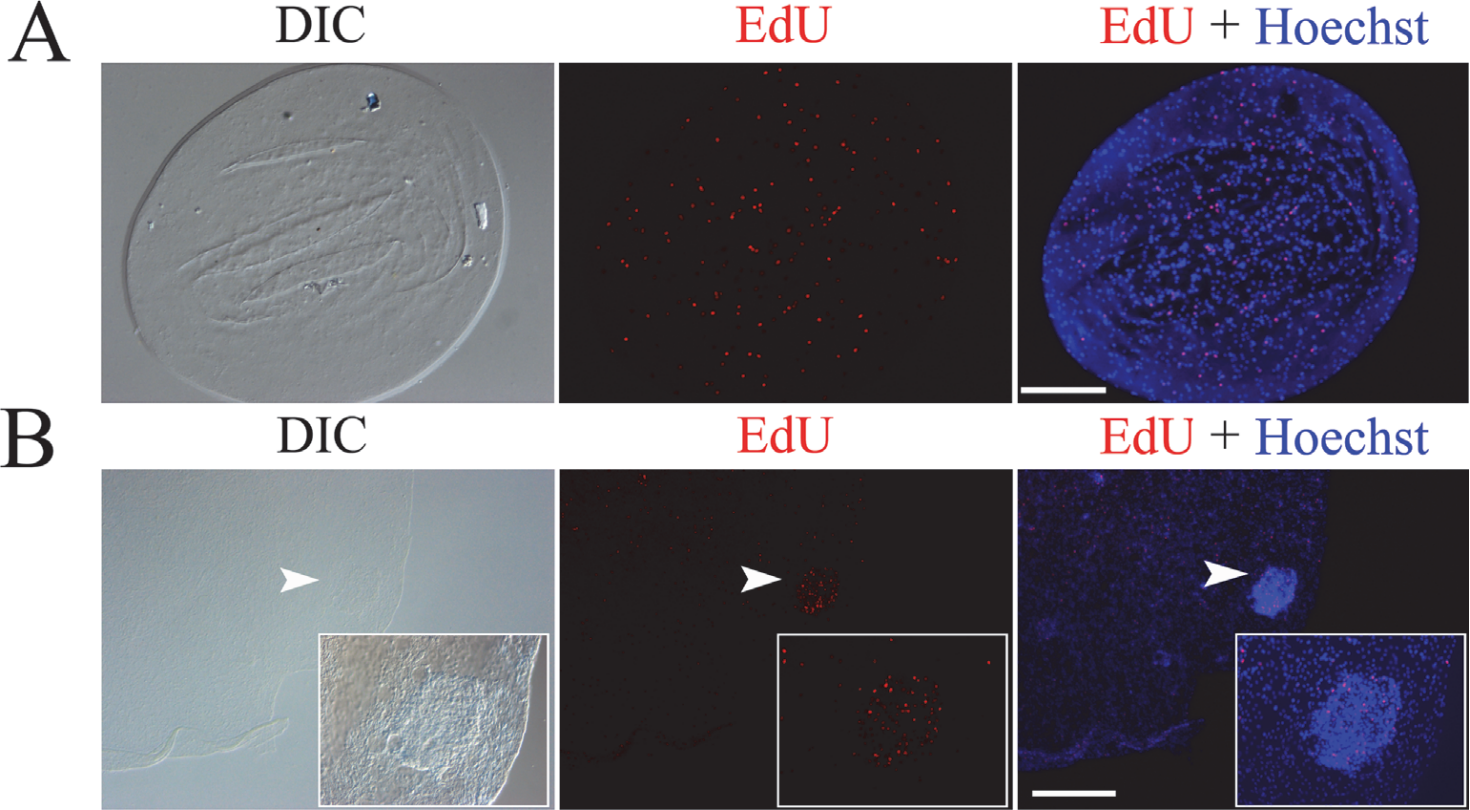
Detection of EdU incorporation in proliferating germinal cells of *in vitro* cultured *E. multilocularis* vesicles. EdU positive cells are shown in a whole small vesicle (A) or in a large vesicle (B). Inserts in (B) are magnified images of a developing protoscolex indicated by the arrow heads. Images of the differential interference contrast (DIC) bright field microscopy are shown. Bar = 125 μm for (A) and 250 μm for (B), respectively.

**Fig 3:**
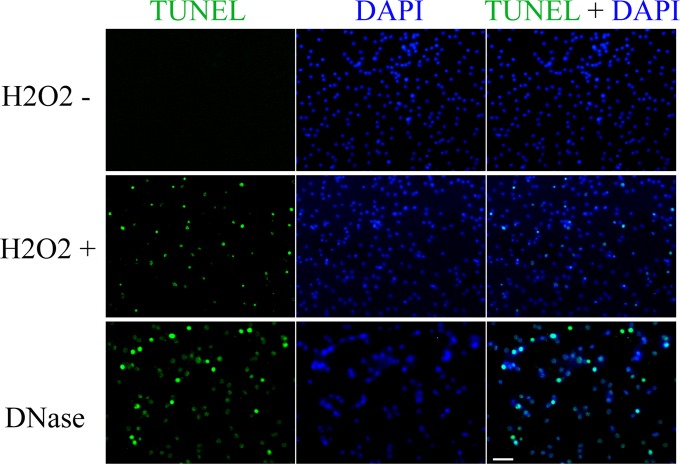
Detection of apoptotic cells by TUNEL in *in vitro* cultured *E. multilocularis* vesicles. Apoptosis was induced by H2O2 treatment (H2O2 +). Note rare apoptosis accrued in the vesicle without H2O2 treatment (H2O2 -). DNase treatment was used as a positive control. Bar = 25 μm.

Analysis of the two vital cellular processes, cell proliferation and cell death, can be achieved by using *in situ* fluorescence analysis of the nucleic acid in the cells. To determine if our method can also be utilized for this, we performed EdU and TUNEL experiments in the GL cells. Detecting the incorporation of EdU is a new method for analyzing DNA synthesis in proliferating cells and has recently been carried out for identify the proliferative cells in *E. multilocularis* [6, 9]. As shown in Fig. 2, we could identify the EdU positive GL cells either in a large vesicle containing developing protoscoleces or in a very small vesicle (about 0.5 mm in diameter), showing the merit of our method in studying on small vesicles. We also successfully detected the oxidative stress-induced apoptosis in the whole-mount prepared *E. multilocularis* metacestode vesicles cultured *in vitro* with TUNEL (Fig. 3). Taken together, these results emphasized the utility of the whole-mount preparations for *in situ* labeling and detecting of nucleic acid in the GL cells.

In conclusion, we expect this rapid and convenient method would facilitate high-resolution microscopic fluorescence analysis of *in vitro* cultivated vesicles. Due to its facility in manipulation of a large amount of vesicles and very small vesicles, we also anticipate this method would be further used for the high-throughput studies (*e*.*g*. drug screen based on the phenotype analysis) and the molecular mechanism studies on *Echinococcus* metacestode vesicle development (*e*.*g*. comparison between the small and large vesicles).

## Supporting Information

S1 ARRIVE Checklist Completed “The ARRIVE Guidelines Checklist” for reporting animal experiments in this manuscript. (DOCX)
