# Supplementary material for: A Rapid and Convenient Method for Fluorescence Analysis of In Vitro Cultivated Metacestode Vesicles from Echinococcus multilocularis
Source: PLoS One. 2015 Feb 23;10(2):e0118215. doi: 10.1371/journal.pone.0118215 (PMC4337908; doi:10.1371/journal.pone.0118215)

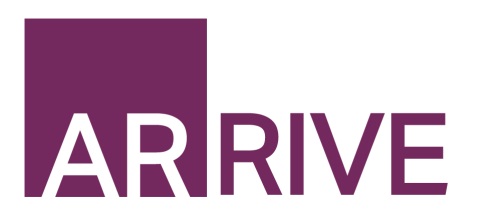


The ARRIVE Guidelines Checklist

Animal Research: Reporting In Vivo Experiments

Carol Kilkenny^1^, William J Browne^2^, Innes C Cuthill^3^, Michael Emerson^4^ and Douglas G Altman^5^

*^1^The National Centre for the Replacement, Refinement and Reduction of Animals in Research, London, UK, ^2^School of Veterinary Science, University of Bristol, Bristol, UK, ^3^School of Biological Sciences, University of Bristol, Bristol, UK, ^4^National Heart and Lung Institute, Imperial College London, UK, ^5^Centre for Statistics in Medicine, University of Oxford, Oxford, UK.*

|  | ITEM | RECOMMENDATION | Section/ Paragraph |
| --- | --- | --- | --- |
| 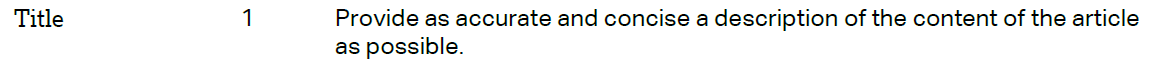 | | | Title |
| 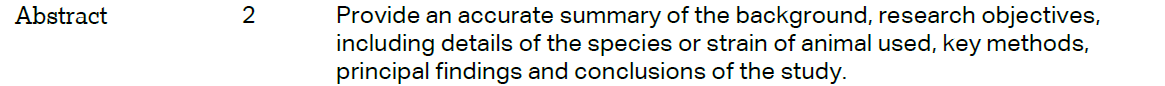 | | | Abstract |
| INTRODUCTION | | |  |
| 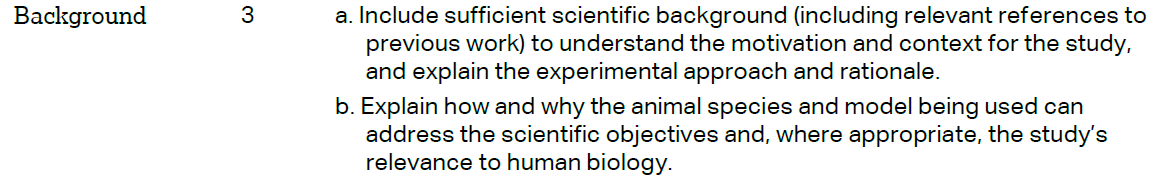 | | | Paragraphs 1-4  Paragraphs 1-2 |
| 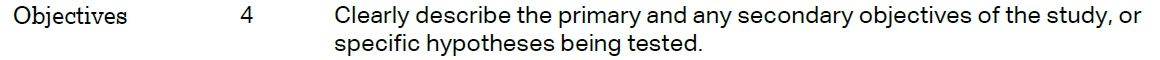 | | | Paragraphs 3-4 |
| METHODS | | |  |
| 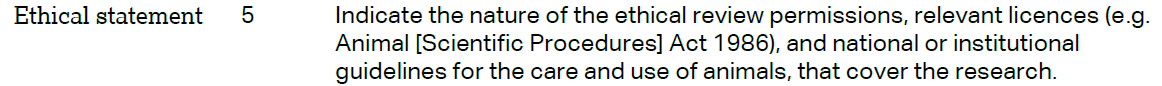 | | | Paragraph 1 |
| 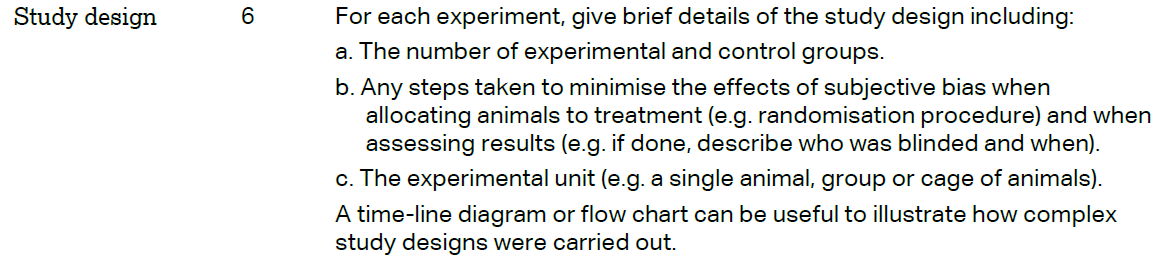 | | | N/A.  Animals used in this study are just for in vivo propagation of the parasite material. |
| 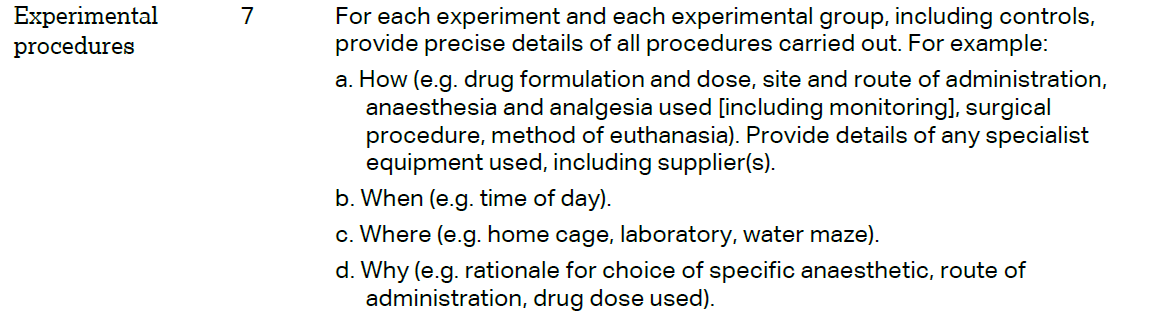 | | | N/A.  The reason is as the same as that in the item 6. |
| 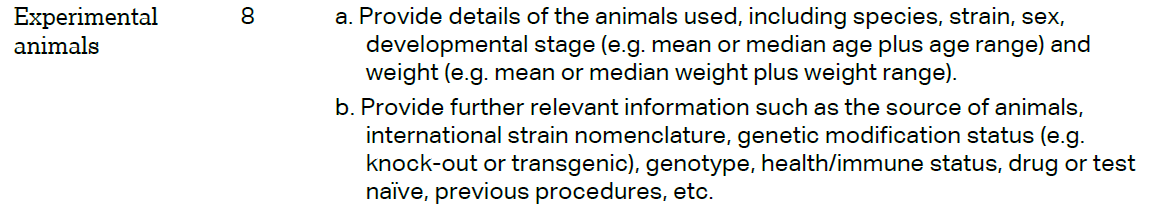 | | | Paragraph 2 |

The ARRIVE guidelines. Originally published in *PLoS Biology*, June 2010^1^

| 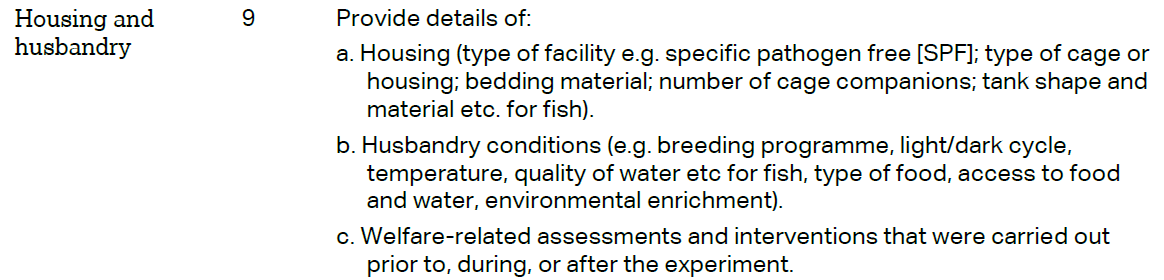 | Paragraphs 1-2 | |
| --- | --- | --- |
| 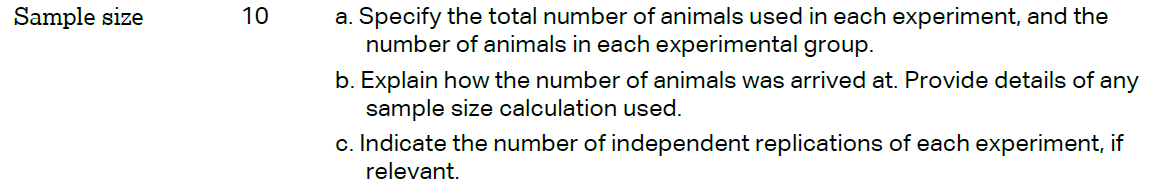 | N/A.  The reason is as the same as that in the item 6. | |
| 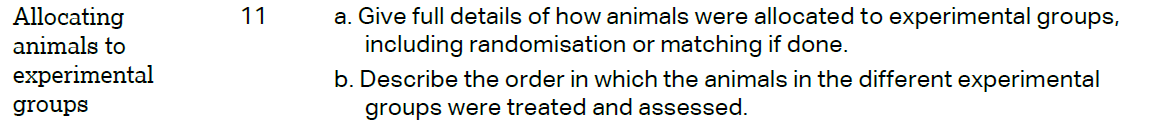 | N/A.  The reason is as the same as that in the item 6. | |
| 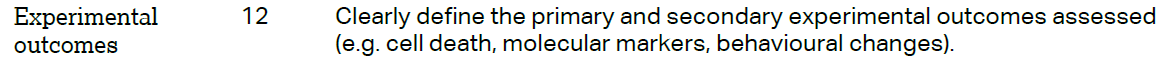 | Paragraphs 5-7 | |
| 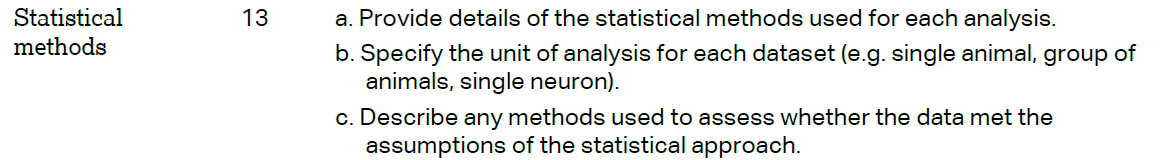 | N/A. We did not use any statistical methods in this study. | |
| RESULTS |  | |
| 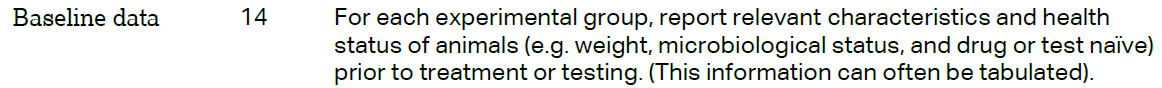 | N/A.  The reason is as the same as that in the item 6. | |
| 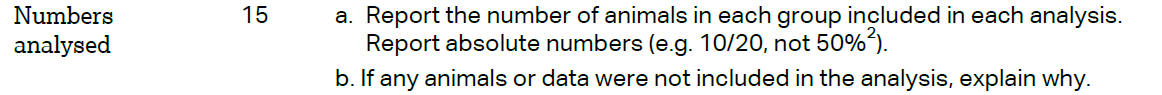 | N/A.  The reason is as the same as that in the item 6. | |
| 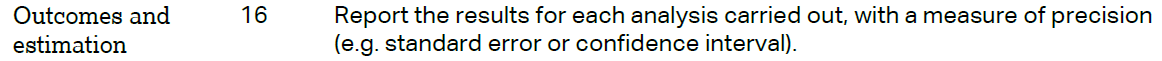 | Paragraphs 5-7. Figures 1-3 | |
| 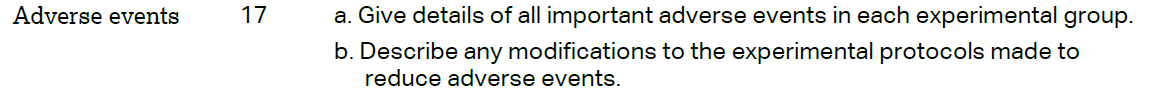 | N/A.  This study is about methodology. | |
| DISCUSSION |  | |
| 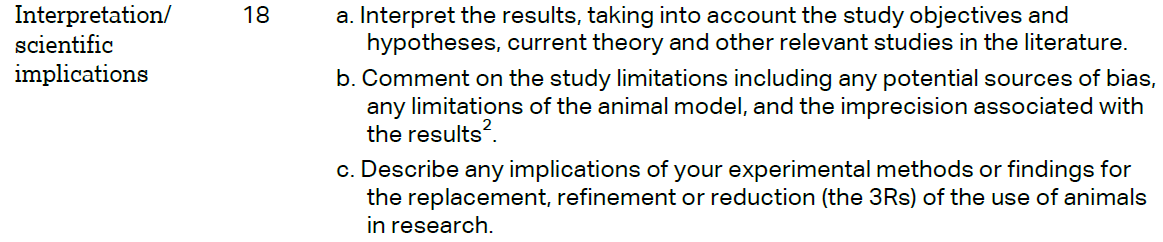 | Paragraphs 1 and 3.  N/A. The reason is as the same as that in the item 6. | |
| 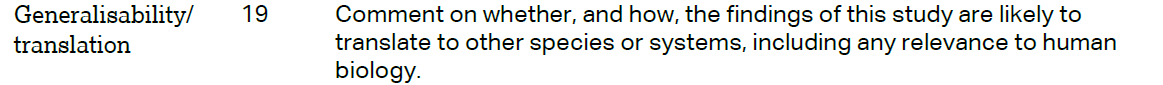 | N/A. The reason is as the same as that in the item 6. | |
| 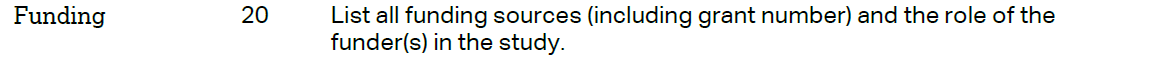 | This is provided on the manuscript submission website of the journal. |  |


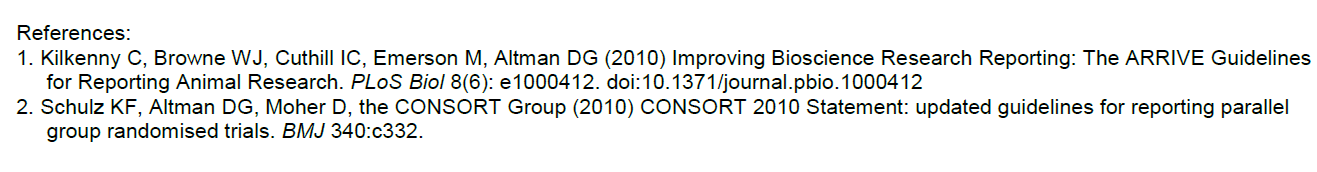

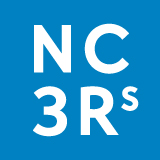

Supplement: S1 ARRIVE Checklist — (DOCX) [file pone.0118215.s001.docx]
